# Expression of selected genes in liver biopsy specimens in relation to early virological response in patients with chronic hepatitis C with HCV mono- and HIV/HCV co-infection

**DOI:** 10.1007/s00705-013-1930-1

**Published:** 2013-12-24

**Authors:** Elżbieta Jabłonowska, Kamila Wójcik, Ewa Koślińska-Berkan, Bożena Szymańska, Aleksandra Omulecka, Anna Piekarska

**Affiliations:** 1 Department of Infectious Diseases and Hepatology, Medical University of Łódź, Kniaziewicza 1/5, 91-347 Łódź, Poland; 2 Central Laboratory of Medical University of Łódź, ul. Mazowiecka 6/8, 92-215 Łódź, Poland; 3 Department of Pathology, Biegański Provincial Specialistic Hospital, Kniaziewicza 1/5, 91-347, Łódź, Poland

## Abstract

The aim of our study was to evaluate the significance of *IL-28B* single-nucleotide polymorphism and hepatic expression of IFI27, SOCS3 and miR-122 in order to predict early virological response (EVR) in patients infected with HCV genotype 1 or 4. The study group consisted of 65 patients: 46 with HCV mono- and 19 with HIV/HCV co-infection. Analyses of *IL-28B* single-nucleotide polymorphism C/T (rs12979860) in the blood and expression of SOCS3, IFI27 and miR-122 in liver biopsy samples obtained before PegIFN and ribavirin treatment were performed by the RT-PCR method. EVR was defined as a >2log decline in HCV viremia at week 12. EVR was associated with a lower expression of IFI27 and a more frequent presence of the *IL28B*CC genotype. IFI27 expression was lower in patients with the CC genotype, irrespective of EVR. In multivariate logistic regression, only *IL28B* CC genotype and age above 40 years influenced EVR (OR =5.09 and 0.29 respectively). In contrast to IFI27, expression of miR-122 and SOCS3 in patients with different IL28B genotypes was not statistically significantly different. A correlation between miR-122 and SOCS3 was found (Rho =0.495094 p< 0.0001). Analysis of IFI27, SOCS3 and miR-122 hepatic expression does not provide substantial benefits for the prognosis of EVR. The only independent prognostic factors for EVR are age and IL28B genotype. The prognostic significance of IFI27 expression for EVR is dependent on the genetic polymorphism of *IL28B*.

## Introduction

Hepatitis C virus infection (HCV) is a global health problem. HCV is a positive-strand RNA virus that infects about 3 % of the world population. According to WHO estimates, 3–4 million new HCV infections occur every year. Approximately 80 % of patients with acute hepatitis C fail to eliminate the virus and become chronically infected. About 20 % of HCV-infected individuals progress to cirrhosis 20 years after acute infection [1–3].

The existing therapy for chronic hepatitis C (CHC) based on pegylated interferon and ribavirin is effective only in half of patients; hence, the introduction of new drugs is crucial. It has been shown that adding protease inhibitors such as boceprevir or telaprevir to this combination can significantly improve the results of the treatment. Administration of such third drug combinations can eradicate HCV in about 70 % of patients with CHC [4, 5]. A triple-drug regimen is especially recommended for patients with pretreatment factors indicating a smaller chance of a good response to Peg-IFN and ribavirin and for patients who have not attained sustained virologic response (SVR) after treatment with pegylated interferon and ribavirin.

The main factors used to predict the response of patients to therapy include HCV genotype, viral load, viral kinetics during treatment, and *IL28B* genotype. Patients who are infected with HCV genotype 1 or 4, have high viral loads, are infected with *IL28B* CT/TT genotypes, or do not undergo a rapid decrease in HCV viremia during the first weeks of therapy have significantly worse treatment results [6–12].

Hepatic expression of interferon (IFN)-stimulated genes (ISGs) is one of the recently recognised prognostic factors for the response to treatment with IFN-α and ribavirin. ISG products are responsible for the antiviral, antiproliferative and immunomodulatory properties of IFN. It appears that lower expression of ISGs in liver biopsy specimens evaluated before therapy corresponds with a better response to treatment [13–15].

Other recently studied prognostic factors include microRNAs (miRNAs), which are well-known posttranscriptional regulators of gene expression. Mir-122, the liver’s most abundant miRNA, has been shown to pair with the genomic RNA of HCV and positively regulate replication of the virus in cell culture [18]. There is evidence that miR-122 expression can be regulated by IFN. Moreover, it is possible that miR-122 can regulate expression of ISGs [19].

The aim of our study was to evaluate the significance of selected clinical parameters – IL-28B polymorphism and expression of IFI27, SOCS3 and miR-122 – in liver biopsy specimens in order to predict early virological response (EVR) in patients infected with HCV genotype 1 or 4.

## Materials and methods

All procedures followed were in accordance with the ethical standards of the responsible committee on human experimentation (institutional and national) and with the Helsinki Declaration of 1975, as revised in 2008. Informed consent was obtained from all patients to be included in the study. Approval from the ethics committee was obtained.

The study group consisted of 65 successive patients, 46 with HCV monoinfection and 19 with HIV/HCV co-infection, who fulfilled the following inclusion criteria:

1. No HBV infection (HBsAg negative)
2. No cirrhosis (in liver biopsy)
3. HCV treatment naive
4. Undetectable HIV viremia (<50 copies/ml) and CD4 count higher than 350 cells/μl in HIV/HCV-coinfected patients on introduction of Peg-IFN-α with ribavirin

The treatment was conducted according to the following protocol: Combined therapy using Peg-IFN-α 2a (Pegasys; Roche, Switzerland) or Peg-IFN-α 2b (PEGIntron; Schering Corp) and ribavirin was applied. Peg-IFN-α was administered subcutaneously once a week in a standard dose (Pegasys dose of 180 μg, PEGIntron dose dependent on the patient’s weight). Ribavirin was administered *per os* daily in a dose dependent on the patient’s weight (less than 75 kg, 1000 mg; above 75 kg, 1200 mg).

Evaluation of HCV viremia was performed in all patients three months after the introduction of the treatment. Patients in whom the viral load was below the detection threshold or declined by more than 2 log were assigned to the group with EVR. The remaining patients were assigned to the group that had not attained EVR.

Before the treatment, a liver biopsy was performed in all patients. The grade of inflammation and necrotic changes and the stage of fibrosis were assessed according to the Batts and Ludwig scale.

In addition to routine tests performed before and during treatment with pegylated IFN and ribavirin, *IL28B* single-nucleotide polymorphism C/T (rs12979860) and hepatic expression of miR-122, IFI27 and SOCS3 were analyzed.

### Analysis of *IL-28B* single-nucleotide polymorphism C/T (rs12979860)

Genomic DNA was isolated from 200 μL of blood using a QIAamp DNA Blood Mini Kit (QIAGEN) according to the manufacturer’s protocol. DNA was quantified using a PicoDrop spectrophotometer (Picodrop Limited). The *IL-28B* single-nucleotide polymorphism C/T (rs12979860) was analyzed using Custom® SNP Genotyping Assays (Applied Biosystems). The primer and probe sequences were as follows: Forward primer, 5’-GCCTGTCGTGTACTGAACCA; reverse primer, 5’-GCGCGGAGTGCAATTCAAC; probe (C allele), 5’-VIC-TGGTTCGCGCCTTC; probe (T allele), 5’-FAM-CTGGTTCACGCCTTC. Genotyping was performed using an ABI7900HT Real-Time PCR System (Applied Biosystems) in 25 μL reaction volume containing 10 ng DNA, a 12.5-μL TaqMan® Universal PCR Master Mix, and 1.25 μL (40x) Custom® SNP Genotyping Assays and analyzed using Sequence Detection System 2.3 software.

### Total RNA isolation

Total RNA was extracted using a mirVana™ miRNA Isolation Kit (Ambion) according to the manufacturer’s instructions. Briefly, frozen samples were homogenized in 300 μl of lysis/binding solution using a TissueRuptor homogenizer (QIAGEN). RNA was eluted in 100 μl of RNase-free water and quantified using a PicoDrop spectrophotometer. The quality of RNA samples was analyzed by measuring the absorbance ratio at 260/280 nm. The purified total RNA was immediately used for cDNA synthesis or stored at −80 °C.

### miRNA-122 expression

Reverse transcription was carried out on 10 ng of total RNA in 15-μl reactions using a TaqMan® MicroRNA Reverse Transcription Kit (Applied Biosystems) according to the manufacturer’s instructions. The RT reaction was diluted 10 times in nuclease-free water, and 9-μL aliquots were subsequently used for PCR amplification using TaqMan® MicroRNA Assays (miR-122 -Assay ID 002130, RNU 24 - Assay ID 001001) according to the manufacturer’s instructions (Applied Biosystems). RNU 24 (SNORD24 small nucleolar RNA, C/D box 24) was used as an endogenous control.

### mRNA expression

*Homo sapiens*-specific TaqMan Gene Expression Assays (Applied Biosystems) for interferon-alpha-inducible protein 27 (IFI27, Hs00271467_m1), suppressor of cytokine signaling 3 (SOCS3, Hs02330328_s1) were used. Generation of cDNA was performed using 250 ng of total RNA with High Capacity cDNA Reverse Transcription Kits according to the protocols of the manufacturer (Applied Biosystems). First-strand cDNA was subsequently diluted 10 times in nuclease-free water before addition to the RT-PCR reaction mixture. mRNA expression levels were analyzed using beta actin (ACTB) as an endogenous control.

### Real-time PCR analysis

#### Real Time PCR analysis

TaqMan PCR assays were performed in 96-well optical plates on a 7900HT Fast Real-Time PCR System (Applied Biosystems) and were analyzed using Sequence Detection System 2.0 software. The cycle threshold of each triplicate determination was normalized by subtraction of the cycle threshold for its corresponding endogenous control (ΔCT). Each ΔCT was then calibrated by subtracting the ΔCT value for control tissue (ΔΔCT). The amount of mRNA was expressed as the logarithm of the relative units calculated from equation 2−ΔΔCT.

#### Statistical methods

Quantitative variables between different groups were compared according to the Mann-Whitney test; and categorical variables, according to chi-square distribution, Yates’ correction for continuity or Fisher’s exact test (depending on the size of the group studied).

Multivariate logistic regression was performed to associate the independent explicative, categorical or continuous variables with dichotomous dependent variable EVR. The backward stepwise model selection procedure was used to sequentially eliminate variables from a model that included IL-28 genotype, expression of SOCS3, IFI27, and miR-122, sex, age and coinfection. To measure the association between two not normally distributed quantities, Kendall’s rank correlation coefficient was applied. Statistical analysis was performed using STATISTICA (data analysis software system), version 10.0, StatSoft, Inc., 2011 (www.statsoft.com). Finally, receiving operator characteristic (ROC) curves were constructed. In ROC curves, the true positive rate (sensitivity) is plotted as a function of the false positive rate (1-specificity) for different cutoff points. Each point on the ROC plot represents a sensitivity/specificity pair corresponding to a particular decision threshold. All p-values reported are two-sided, and p-values<0.05 are considered significant. Statistical analysis was performed using the computing environment R, version 2.15.2 (The R Foundation for Statistical Computing).

## Results

### Characteristics of patients divided into two groups according to EVR

Patients in these two groups were well matched for most clinical variables, with the exception of IFI27 and *IL28B* gene polymorphism expression (Table 1). Lower expression of IFI27 and more frequent presence of *IL28B* CC genotype were observed in the group with EVR (Table 1).

**Table 1:** Characteristics of the study group according to EVR

	Without EVR (N=19)	With EVR (N=46)	Significance
ALT>50 (U/L)	14 (73.7 %)	27 (58.7 %)	NS
IL28 B genotype:			
CT	13 (68.4 %)	21 (45.6 %)	NS
TT	3 (15.8 %)	5 (10.9 %)	NS
CC	3 (15.8 %)	20 (43.5 %)	P=0.046
Women	5 (26.3 %)	11 (23.9 %)	NS
Age>40 years	8 (42.1 %)	9 (19.6 %)	NS
HIV/HCV	5 (26.3 %)	13 (28.3 %)	NS
G>=2	7 (36.8 %)	11 (23.9 %)	NS
S>=2	15 (78.9 %)	32 (69.6 %)	NS
HCV genotype:			
1	15 (78.9 %)	39 (84.8) %	NS
4	4 (21.1 %)	7 (15.2 %)	NS
HCV viremia >6 × 10^5^ (IU/mL)	13 (68.4 %)	27 (58.7 %)	NS
ALT (U/L)	72.0 (50.0-91.0)	53.5 (39.0-83.0)	NS
Age (years)	36.0 (21.0-51.0)	30.5 (22.0-37.0)	NS
HCV wiremia (× 10^6^ IU/mL)	4.1 (0.6-8.9)	1.6 (0.4-5.1)	NS
IFI27	28.2 (5.8-50.8)	5.7 (2.4-24.2)	P=0.011
SOCS3	3.1 (2.4-15.2)	5.4 (1.7-10.9)	NS
MiR-122	2.0 (0.9-2.8)	3.0 (1.2-4.1)	NS

Data are presented as a percentage (%) or median and interquartile range: median (25 %-75 %)

EVR, early virological response

In liver biopsy: G, grade of inflammation and necrosis; S, stage of fibrosis; NS, not significant

### *IL28B* polymorphism and IFI27 expression are not independent predictors of the response to treatment

**Fig. 1:**
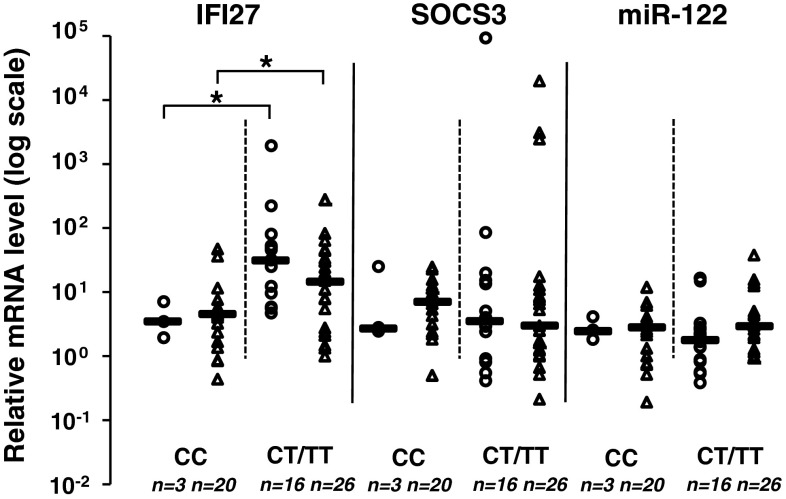
Relationship between IL28B genotype and IFI27, SOCS3 and miR-122 mRNA expression in liver tissue in patients with and without early virological response (EVR). Gene expression analysis by relative quantitative (RQ) real-time PCR. mRNA values reported as logarithm of RQ were normalized to ACTB for IFI27 and SOCS3 and to RNU24 for miR-122. Circles indicate values in patients with EVR; triangles, without EVR. *Statistically significant difference

We found a statistically significant association between *IL28 B* allele variants and the expression level of IFI27. As expected, the *IL28B* CC genotype (which is associated with a better response to treatment) was correlated with lower expression of IFI27. IFI27 expression was lower in patients with the CC genotype, irrespective of EVR (Fig. 1). Patients with EVR and the CC genotype had lower IFI27 expression compared to patients with EVR and the *IL28B* CT/TT genotype (p=0.0244). Patients without EVR but with the *IL28B* CC genotype had lower IFI27 expression than patients without EVR but with the CT/TT genotype (p=0.0323). If IFI27 expression affected EVR independently of *IL28B* polymorphisms, subjects with EVR would have lower expression of IFI27 regardless of *IL28B* genotype, which was not established in our study.

In contrast to IFI27, expression of miR-122 and SOCS3 in patients with different *IL28B* genotypes was not statistically significantly different. The lack of statistical significance was observed regardless of the type of EVR (Fig. 1).

### Independent predictors of EVR include age of the patient (over 40 years) and *IL28B* genotype. MiR122 does not appear to be a relevant predictive factor for EVR

Results of multivariate logistic regression showed that expression of any of the analyzed genes was not an independent factor affecting EVR. Such factors included age of patients and *IL28B* polymorphism (Table 2).

**Table 2:** Prognostic factors for EVR: analysis of multivariate logistic regression using the Rosenbrock and quasi-Newton method

	Constant B0	*IL28B* CC genotype	Age >40 years
Regression coefficient	0.83	1.63	−1.34
Standard error	0.37	0.74	0.65
t(62)	2.23	2.20	−2.06
P	0.0294	0.0313	0.0433
−95 %CL	0.09	0.15	−2.65
+95 %CL	1.57	3.10	−0.04
Chi-square Wald	4.97	4.85	4.25
P	0.0258	0.0276	0.0391
Odds ratio for a one-unit change	2.29	5.09	0.26
−95 %CL	1.09	1.16	0.07
+95 %CL	4.80	22.30	0.96
Odds ratios range		5.09	0.26
−95 %CL		1.16	0.07
+95 %CL		22.3	0.96

The parameter that were obtained indicate that the presence of the CC genotype increases the likelihood of early virological response (EVR). Age >40 reduces the likelihood of EVR

The above table contains the parameters of regression analysis. Regression coefficients are the most important parameter among the ones listed. Based on this parameter, we can determine how CC genotype and age above 40 years influence the likelihood of EVR

The risk of EVR in patients with CC genotype at an age above 40 years was 0.26 times lower than in subjects with CC genotype under the age of 40 years. However, the risk of EVR in the individuals aged over 40 years with the CC genotype was 5.09 times higher than in those aged over 40 years with a genotype other than CC.

**Fig. 2:**
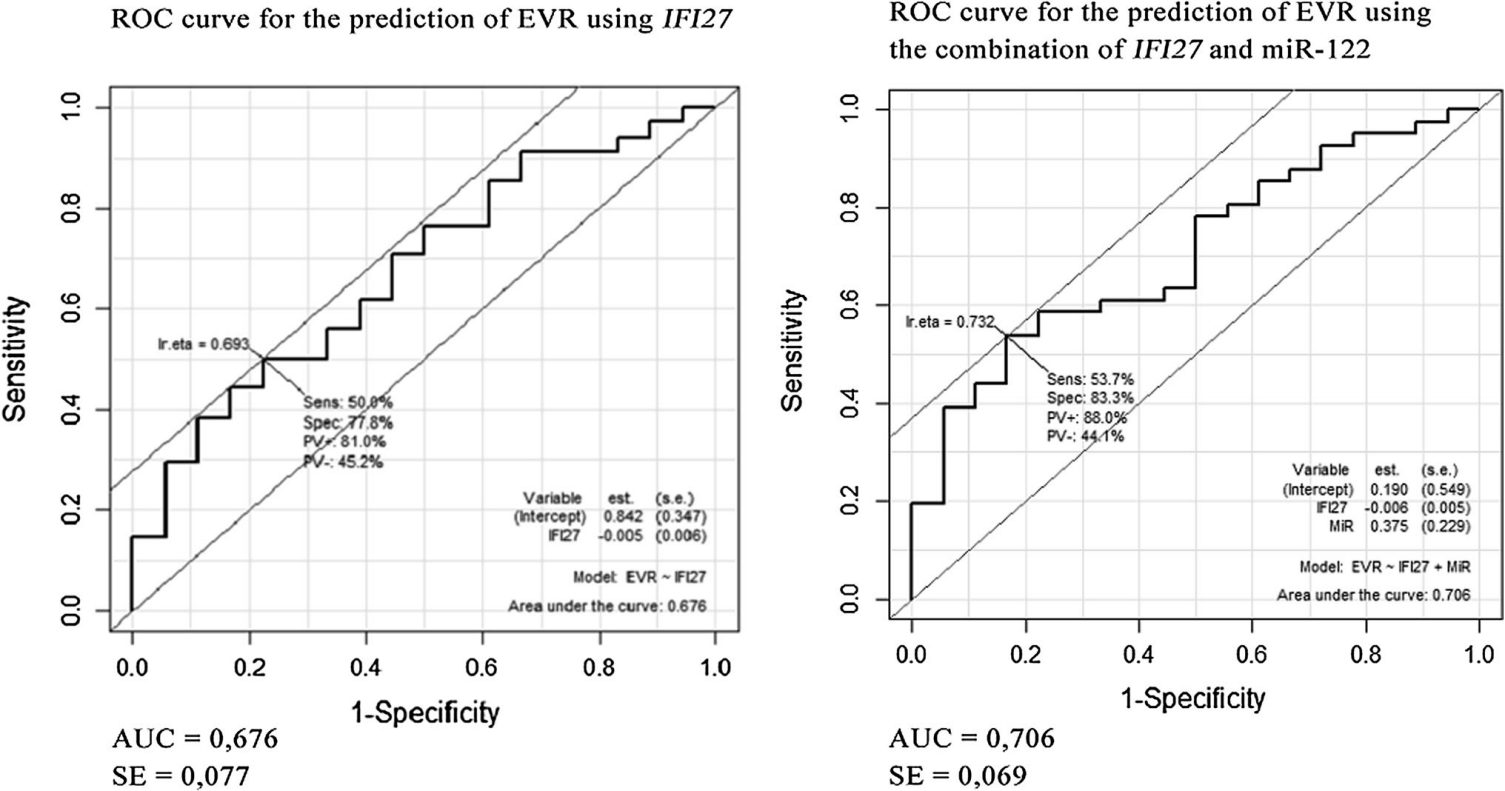
Adding the evaluation of miR-122 expression to the analysis of IF127 expression did not significantly influence the prediction of EVR

Adding the evaluation of miR-122 expression to the analysis of IFI27 expression did not significantly influence the prediction of EVR, as shown in Fig. 2

.

### Correlations between miR-122 and the analyzed genes

No correlations between miR-122 and IFI27 were observed. (Rho = 0.114845).

A correlation between miR-122 and SOCS3 was found (Rho = 0.495094 p< 0.0001).

## Discussion

An efficient interferon system is required for controlling viral infections, and up to now, IFN-α has remained an essential part of anti-HCV treatment.

Counterintuitively, preactivation of the endogenous IFN system, observed as an increased expression of hepatic ISGs before treatment of patients with CHC, is associated with less frequent achievement of SVR to Peg-IFN and ribavirin therapy [13–15]. Our results confirm these observations. Patients who had not achieved EVR had higher expression of IFI27 (alpha-inducible protein 27), a gene from the group of ISGs, which have been shown in numerous studies to be of great prognostic importance [15, 20, 21].

The mechanisms causing the induction of ISGs in the liver of patients with poor response to IFN-α are not understood. It is possible that genetic factors are responsible for the discrepant expression of ISGs, which can also correspond to different responses to IFN therapy. Interestingly, a single-nucleotide polymorphism (SNP) near the *IL28B* gene can predict the response to hepatitis C treatment with interferon and ribavirin [6, 9–11]. The results of our study confirmed this hypothesis, inasmuch as we found that the expression of IFI27 is associated with the genetic polymorphism *IL28B*. IFI27 expression was lower in patients with the CC genotype, irrespective of EVR, which suggests that that *IL28B* genotype determines IFI27 expression.

SOCS3 is a protein that operates through a negative feedback curve. Overexpression of this protein inhibits the initial signal responsible for its own induction by blocking the Jak/STAT pathway. Huang et al. [22] demonstrated that SOCS3 expression was 11 times higher in patients whose HCV viral load decreased by 2 log in the fourth week of treatment compared to the individuals with no decline in viremia. Conversely, in the study presented by Persico et al. [23], a worse response to antiviral treatment was observed in patients with an increased expression of the gene encoding this protein. In our study, no statistically significant difference between SOCS3 expression in patients with and without EVR was observed. Expression of SOCS3 had no influence on EVR. Discordant results presented by other authors indicate the lack of practical significance of the analysis the expression of this gene for predicting the results of antiviral therapy.

The relationship between HCV and miR-122 is complex. On one hand, miR-122 can interact with HCV and positively regulate replication of the virus in cell culture [18]. On the other hand, as presented by Pedersen et al. [19], miR-122 expression can be negatively regulated by IFN in Huh-7 cells.

According to our results, miR-122 expression was not a relevant prognostic factor for the response to treatment with IFN and ribavirin. In our study, a higher level of expression of miR-122 was observed in patients with EVR in comparison to those without EVR, but this difference was not statistically significant. Adding the evaluation of miR-122 expression to the analysis of IFI27 expression did not significantly increase the predictive value of the latter. However, the literature presents several contradicting opinions on this subject. It was demonstrated by some authors that lower levels of miR-122 were associated with a worse response to treatment with IFN and ribavirin [16, 17]. In the study conducted by Murakami et al. [24] miR-122 expression with FDR correction did not differ statistically significantly in patients with SVR compared to those who did not attain SVR.

Interestingly, in a study conducted *in vitro* by Yoshikawa et al. [25], silencing of miR-122 function enhanced IFN-induced interferon-stimulated response element (ISRE) activity by decreasing expression of SOCS3.

In our study, no correlation between miR-122 expression and either *IL28B* genotype or IFI27 expression was found. However, we observed a positive correlation between miR-122 and SOCS3. Despite numerous studies on this subject, the role of miR-122 in the pathogenesis of HCV requires further investigation.

Presently, as non-invasive methods for diagnosing fibrosis in patients with chronic viral hepatitis C, such as fibroscan or elastometry, gradually replace liver biopsy, performing this procedure with a view to analyzing IFI27, SOCS3 and miR-122 expression for the prognosis of EVR is not associated with additional benefits.

## Conclusions

Analysis of hepatic expression of IFI27, SOCS3 and miR-122 does not provide substantial benefits for the prognosis of EVR. The only independent prognostic factors for EVR are age and IL28B genotype. The prognostic significance of IFI27 expression for EVR is dependent on the genetic polymorphism of *IL28B*.
